# The Effect of *Moringa oleifera* Leaf Powder on the Physical Quality, Nutritional Composition and Consumer Acceptability of White and Brown Breads

**DOI:** 10.3390/foods9121910

**Published:** 2020-12-21

**Authors:** Laurencia Govender, Muthulisi Siwela

**Affiliations:** Discipline of Dietetics and Human Nutrition, School of Agricultural, Earth and Environmental Sciences, University of KwaZulu-Natal, Private Bag X01, Scottsville 3209, Pietermaritzburg 3201, South Africa; GovenderL3@ukzn.ac.za

**Keywords:** *Moringa oleifera* leaf powder (MOLP), bread, fortification, nutritional composition, consumer acceptability

## Abstract

Fortifying popular, relatively affordable, but nutrient-limited staple foods, such as bread, with *Moringa oleifera* leaf powder (MOLP), could contribute significantly to addressing under nutrition, especially protein and mineral deficiencies, which are particularly prevalent among a large proportion of populations in sub-Saharan African countries. The current study aimed to determine the effect of MOLP on the physical quality, nutritional composition and consumer acceptability of white and brown breads. The texture, colour and nutritional composition of white and brown bread samples substituted with 5% and 10% (*w*/*w*) MOLPs were analysed using standard methods and compared with the control (0% MOLP). A consumer panel evaluated the acceptability of the bread samples using a nine-point hedonic scale. Bread samples became darker as the concentration of MOLP was increased, whilst nutrient levels increased. The overall consumer acceptability of the bread samples decreased with increasing concentrations of MOLP. However, brown bread samples were significantly more acceptable compared with corresponding white bread samples (*p* < 0.05). Under the experiment conditions of the current study, it seems that the bread containing 5% MOLP can be used to contribute significantly to addressing malnutrition, with respect to protein deficiency.

## 1. Introduction

Food insecurity and malnutrition are significant problems globally, specifically undernutrition (wasting, stunting, underweight and micronutrient deficiencies) and hunger [1]. Moderate and severe hunger affects approximately two billion individuals worldwide [1], which contributes significantly to the high rates of malnutrition seen in the world. Approximately 21.3%, 6.9% and 5.6% of children globally are stunted, wasted and overweight, respectively. Additionally, the 2020 nutrition global report indicates that one in nine and one in three individuals are hungry or malnourished and overweight or obese, respectively [2]. The COVID-19 pandemic has contributed to the increase in undernutrition, especially in countries where people are facing financial difficulties [1]. Monotonous diets consisting of largely starchy staple foods are mostly consumed in low- to middle-income countries, such as South Africa (SA). Further, the majority of communities from these countries consume limited amounts of fruits and vegetables and animal source foods [1]. Animal source foods are high in quality protein, but are less affordable to many impoverished households in SA and other developing countries, compared to plant-based protein sources [3]. This type of diet also lacks dietary diversity and could also lead to micronutrient deficiencies. Micronutrient deficiencies are a public health concern, especially in developing countries such as SA. Vitamin A, iron and zinc deficiencies are particularly problematic [4]. One of the main reasons for the trends of an increase in poor diets is the rising food costs. A basic 28-item food basket in SA in 2020 costs approximately USD 53.69 [3], which can be unaffordable to many, especially those that are hard hit by the current economic situation and global pandemic. The poorest are the most affected and are at significant risk for food insecurity [5]. Over the years, the consumption of indigenous crops such as *Moringa oleifera* has decreased due to more Westernised cultures being adopted [6]. This has resulted in less dietary diversity as foods from the formal markets (supermarkets, etc.) are expensive. Indigenous crops, such as *Moringa oleifera,* are known to be nutrient-rich as well as have many health beneficial properties. *Moringa oleifera* is one of 13 species of the *Moringaceae* family of plants and is widely researched. This plant originated in India and Africa but is now widely grown in other parts of the world [7]. Not only can *Moringa oleifera* thrive under different climatic conditions—i.e., in tropical and subtropical countries—it also has nutritional, antioxidant and phytochemical benefits [8].

Furthermore, Moringa is a good source of iron, which is generally deficient in most leading staple plant-based diets such as the starchy staples [9]. Iron is an important micronutrient, especially during pregnancy as it contributes to foetal growth. A pregnant woman who has iron-deficiency anaemia is at great risk for perinatal and maternal mortality, premature delivery and having a low birth weight infant [10,11]. *Moringa oliefera* is also rich in vitamins such as the provitamin A beta-carotene, folic acid, pyridoxine and nicotinic acids and vitamins C, D and E [12]. Vitamin A deficiency is also common in SA and most other developing countries, especially in the sub-Saharan African region. The human body needs vitamin A, and its deficiency affects vision, growth, development, protein synthesis and could result in a child not being able to reach their full potential, both physically and mentally [13]. *Moringa oleifera* is also a good source of protein and contains 16–19 amino acids. Ten of these amino acids are essential [14]. Wasting can present in the form of protein-energy malnutrition (PEM) and is caused by a deficiency of good quality protein in the diet [15]. When an individual is malnourished, their body goes into a state of starvation and negatively affects the immune system, kidneys, cardiac muscle, liver and gastrointestinal tract [13,15]. Micronutrient deficiencies and PEM are commonly seen in vulnerable population groups, such as of a woman of childbearing age and children under five years. These groups are thus targeted for nutritional interventions.

Staple foods such as bread are commonly consumed food items in SA [16], and since October 2003, wheat flour fortification was made mandatory [17]. However, access to fortified foods still remains challenging to many impoverished individuals, as many of these individuals rely on social grants to purchase food [3,18,19]. Baked bread contains high amounts of energy, carbohydrates and fat, but is limited in other nutrients such as protein, minerals and vitamins [20]. To increase the nutritional composition of bread, *Moringa oleifera* leaf powder (MOLP) could be used as it is rich in proteins and several micronutrients that are deficient in bread. Bread is an affordable source of energy (in the form of starch) and, therefore, would be a suitable candidate for supplementation with MOLP [21]. Several studies have investigated the nutritional composition and consumer acceptability of bread fortified with MOLP (Table 1). Consumers living in different geographical locations (e.g., countries) may show different preference and acceptability levels for the same innovative food product developed from the same conventional product. Thus, there may be differences in consumer acceptability of a MOLP fortified bread across different countries. While studies conducted in other countries found that the dark colour and bitter taste of MOLP impacted negatively on consumer acceptability of bread [22,23], different results may be obtained with consumers living in SA. To the best of the researchers’ knowledge, this study is first to investigate consumer acceptability of a MOLP fortified bread in SA. Brown bread is dark in colour, so it may be a suitable food item for supplementing with MOLP as its dark colour might not significantly change due to the addition of MOLP, which is also dark in colour. It seems none of the previous studies listed in Table 1 compared consumer acceptable of corresponding samples of white and brown breads supplemented with MOLP. Furthermore, the other authors from earlier studies [23,24,25,26,27,28] (Table 1) did not compare the nutritional composition obtained from their respective study to the estimated average intake values. Consequently, the authors did not determine the exact amount of nutrients that would be obtained from the consumption of usual portions of MOLP fortified bread. Therefore, this study determined the effect of MOLP on the physical quality, nutritional composition and consumer acceptability of white and brown breads in SA.

## 2. Methodology

### 2.1. Study Design

**Figure 1:**
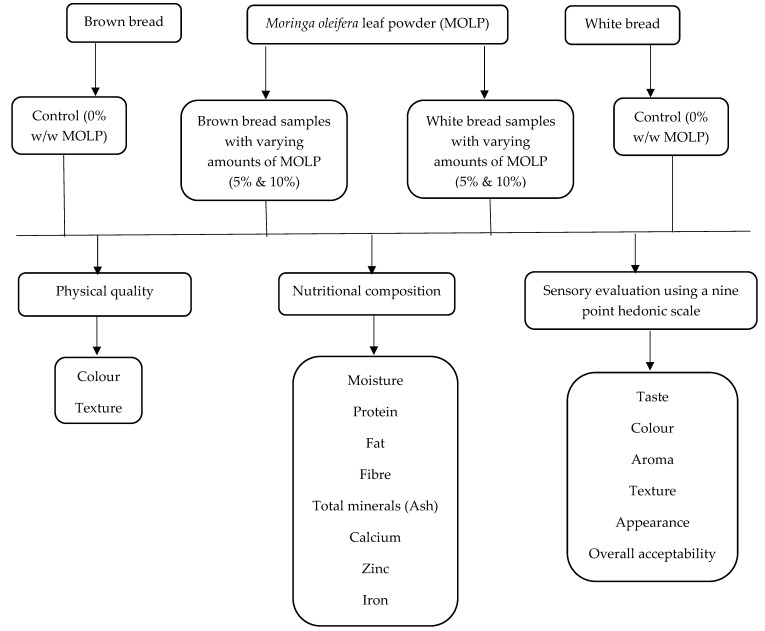
Conceptual framework of the methodology.

This study was a cross-sectional experimental design. Figure 1 presents a conceptual framework of the methodology.

### 2.2. Preparation of Bread

*Moringa oleifera* leaf powder was purchased from a local pharmacy in Pietermaritzburg, SA. The bread (both white and brown) was prepared using a standardised bread making recipe [29]. *Moringa oleifera* leaf powder partially replaced wheat flour at 5% and 10% substitution levels of MOLP in both white and brown breads, respectively. Standard white and brown bread types served as corresponding controls (0% MOLP) for the white and brown bread samples, respectively. Fresh bread samples were baked on the day of data collection.

#### 2.2.1. Ingredients

In this experiment, the ingredients listed below were used.

180–250 mL lukewarm water;
300 g brown/white bread flour;
10 g dry yeast;
3.8 g salt;
15 mL melted butter;
15 g or 30 g MOLP for substitution (This will be added after the flour and salt is sifted).

#### 2.2.2. Method

The method for making bread involved the sequential steps described next. Sift flour and salt into a large bowl. Make a well in the centre of the flour in the large bowl. In a small bowl, mix the dry yeast with 60 mL water and stir until dissolved. Pour yeast mixture into the well of flour and add warm melted butter and 180 mL of water. Mix the ingredients with your fingers, then beat with your hands, adding a little more water if necessary, to make a firm dough. Using your hands, fold and slap the dough against the side of the bowl until it starts to feel elastic and leaves the sides of the bowl. Place the dough onto a floured work surface and knead by folding the far edge towards you, then pushing it firmly away with the heel of the hand. Turn the dough a little and repeat. Continue kneading until the dough is smooth and elastic and springs back when you make a dent with your finger. Place the dough in a clean, warm, oiled bowl, turn it over so that the dough is slightly oiled all over, then cover it with oiled plastic wrap and a cloth. Leave to rise in a warm place for 1 h. After the allocated time, test by pushing a finger into the dough—if the indent remains, it is ready to knock back the dough by punching with your fist several times, squeezing out any large bubbles. Place onto a lightly floured surface and knead three to four times. Pat the dough until round, then fold sides under to make a neat oblong. Press together and seal, then place in a lightly oiled 23 × 12 cm bread tin. Cover loosely with oiled plastic wrap and a cloth, leave in a warm place until it rises to the top of the tin. Preheat the Defy Thermofan Stove (Model 731 MF) oven to 230 °C. Bake the bread at 230 °C for 15 min, then turn the tins around, reduce to moderate heat, i.e., 180 °C, and bake for a further 20–25 min. The bread is cooked if it sounds hollow when the underside is knocked with the knuckles. Take the bread out of tins and leave to cool on a wire rack.

### 2.3. Physical Quality

A Hunter Lab colourimeter was used to measure the colour in all six bread samples. The L measured lightness, a measured redness/greenness and b measured yellowness/blueness. A TA-XT2 Plus texture analyser was used to determine the texture of the bread samples [30]. The texture probe analyser (TPA) applied a force to the bread sample at a speed of 2.0 mm/s, and the force compression was recorded.

### 2.4. Nutritional Composition

The nutrition analysis was conducted on all six bread samples in duplicate for moisture, protein, fat, fibre (NDF), total mineral (ash), calcium, iron and zinc using standard methods [31].

#### 2.4.1. Moisture

The moisture content of all six bread samples was determined using the Association of Official Analytical Chemists (AOAC) Official Method 934.01 [31]. The bread samples were dried in an air circulated oven at 90 °C for 72 h. The moisture content was then calculated using the weight loss content of the samples.

#### 2.4.2. Protein

The protein content was measured with a LECO Truspec Nitrogen Analyser, using the AOAC Official Method 990.03 [31]. The six bread samples were individually placed in a combustion chamber with an autoloader at 950 °C. The percentage protein content was calculated.

#### 2.4.3. Fat

The fat content of the six bread samples was determined according to the AOAC Official Method 920.39 [31], following the Soxhlet procedure. A Büchi 810 Soxhlett Fat Extractor was used to extract the fat in petroleum ether which was then used to calculate the percentage fat.

#### 2.4.4. Fibre (NDF)

The fibre content of the bread samples was measured as Neutral Detergent Fibre (NDF). The NDF was determined according to the AOAC Official Method 978.10, using the Dosi-fibre system [31].

#### 2.4.5. Total Mineral Content (Ash)

The ash content, otherwise known as the total mineral content, was determined using the AOAC Official Method 942.05 [31]. The bread samples were placed in a furnace and heated at 550 °C for 72 h.

#### 2.4.6. Calcium, Zinc and Iron

The calcium (Ca), zinc (Zn) and iron (Fe) contents were determined according to the AOAC Official Method. The bread samples were dried at 105 °C for 2 h, then ashed in a furnace at 550 °C for 4 h. Deionized water and hydrochloric acid (HCl) were added to the ash and boiled in a water bath until dry; this was repeated twice to allow minerals to be drawn into the solution. After 24 h, an atomic absorption spectrophotometer was used to measure the Ca, Zn and Fe contents.

### 2.5. Sensory Evaluation

Fifty-four students and staff members from the agricultural campus of the University of KwaZulu-Natal (UKZN) were recruited to participate in the study. A pilot study was conducted before the main study using 10 participants to test the acceptability of the recipes and adjust the methods accordingly. In the pilot study, 0%, 10% and 20% (*w*/*w*) substitutions were used; however, the 20% substitution was not well accepted. Therefore, the MOLP substitution levels were adjusted to 0%, 5% and 10% (*w*/*w*) for the main study. The pilot study participants were not allowed to participate in the main study. To prevent participants from communicating with one another, they were placed in separate cubicles. Each of the six samples was assigned a unique three-digit code obtained by a table of random numbers, and the tables of random permutations of nine were used to determine the serving order [32]. Each participant was given about a quarter of a slice of each of the six bread samples on a polystyrene plate. Each panellist was given a cup of water so that they could rinse their palate between tasting each bread sample and the sensory evaluation questionnaire. The questionnaire provided was in English, as this is the language of instruction at UKZN. The questionnaire made use of the nine-point hedonic scale (1 = dislike extremely to 9 = like extremely) together with the sensory attributes (taste, colour, aroma, texture, appearance and overall acceptability), which was explained to the panellist before the commencement of the sensory evaluation. Participants were given limited information on MOLP so that bias was prevented. Research assistants helped panellists, if necessary, during the sensory evaluation.

### 2.6. Ethical Consideration

Ethical approval was obtained from the UKZN Humanities and Social Science Ethics Committee (HSS/1244/015D). The gatekeeper’s permission was obtained from the registrar of UKZN. All panellists were required to sign a written consent form before participating in the study.

### 2.7. Statistical Analysis

Data from the physical quality, nutritional composition and sensory evaluation questionnaires were entered into an Excel spreadsheet and cross-checked for accuracy. Thereafter, data were transferred to the Statistical Package for Social Science^®^ (SPSS) version 25 (IBM Corp., Armonk, NY, USA) for analysis. Appropriate statistical techniques, including the Bonferroni and Tukey tests, were used to analyse the data. A *p*-value of <0.05 was considered to be statistically significant.

## 3. Results

### 3.1. The Effect of Moringa oleifera Leaf Powder on the Physical Quality of White and Brown Breads

**Figure 2:**
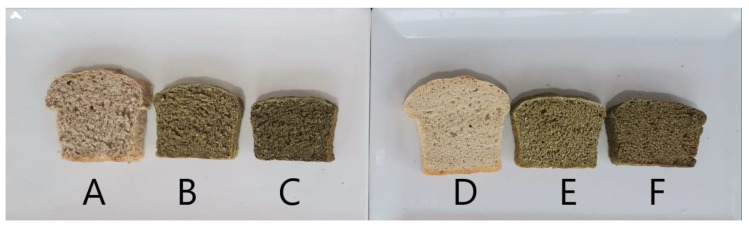
Depicts the different bread samples ((**A**): Brown bread (control, 0%); (**B**): Brown bread (5%) (**C**) *Moringa oleifera* leaf powder [MOLP]); Brown bread (10% MOLP); (**D**): White bread (Control, 0%); (**E**): White bread (5% MOLP); (**F**): White bread (10% MOLP)).

Figure 2 shows the six bread samples, and Table 2 presents the effect of MOLP on the colour and texture of the bread.

The Bonferroni test indicated that there was no significant difference between both control bread and bread containing 5% and 10% MOLPs. However, there was a significant decrease in the lightness of bread with the addition of MOLP. White bread containing 10% MOLP had a darker colour in comparison to brown bread containing 10% MOLP (Figure 2 and Table 2). There was no effect on the texture of the bread samples with the addition of MOLP at different substitution levels.

### 3.2. The Effect of MOLP on the Nutritional Composition of White and Brown Breads

Table 3 presents the nutritional composition of white and brown breads containing MOLP.

The Tukey test indicated that the total mineral content in white bread significantly increased when MOLP was added. There was a slightly significant increase in the total mineral content when 5% MOLP was added to brown bread, and there was a significant increase in brown bread with 10% MOLP (*p* < 0.05). A significant increase in protein content was seen in white bread containing 5% and 10% MOLPs. Further, as the MOLP concentration increased in white bread, so too did the protein content (*p* < 0.05). There was a slightly significant increase in the protein content when 5% MOLP was added to brown bread, and there was a significant increase in brown bread with 10% MOLP (*p* < 0.05). White bread containing MOLP has a higher protein content than brown bread containing MOLP. Addition of MOLP to a concentration of 10% in white bread and 5% in brown bread would result in a significant increase in the iron contents of the two bread types, respectively.

#### Nutritional Composition of White and Brown Breads Compared to the Estimated Average Requirement

The nutritional composition of the bread was compared to the estimated average requirement (EAR) for protein and iron for vulnerable population groups (children under five years and women of childbearing age). The EAR value is one of the four dietary reference intake values and presents the daily average nutrient intake for particular nutrients for specific gender and age groups [33]. In the current study, an estimated weight for a child aged 1–3 years was 13 kg, 4–5 years was 24 kg, 14–18 years was 55 kg and 19–50 years was 62 kg. A standard size of bread is 30 g. A child aged 1–3 years consumes ½ a slice of bread three times a day, a child 4–6 years consumes approximately one slice of bread three times a day, a female aged 14–18 years consumes two slices of bread three times a day and a female adult aged 19–50 years consumes three slices of bread three times a day. Table 4 and Table 5 present the percentage of the EAR for protein and iron, respectively, that would be met from the consumption of estimated bread portions by the respective vulnerable population groups.

### 3.3. The Effect of MOLP on the Sensory Acceptability of White and Brown Breads

Fifty-four students and staff members from the UKZN agricultural campus were recruited to participate in the study. Table 6 indicates the effect of MOLP on the overall acceptability of white and brown breads, as indicated by the percentage distribution of the sensory evaluation scores.

Table 6 shows that as MOLP was increased in either white or brown bread at different substitution levels (5% and 10%), the overall acceptability decreased. However, in terms of the addition of MOLP to bread, there was a less negative effect when MOLP was added to brown bread at different substitution levels in comparison to white bread. Table 7 indicates the effect of MOLP on taste, colour, aroma, texture, appearance and overall acceptability of both white and brown breads.

The Tukey test indicated that there was a significant decrease in taste acceptability when MOLP was added to both white and brown breads, respectively (*p* < 0.05). However, unlike white bread which showed no significant difference in taste acceptability with the addition of either 5% or 10% MOLP, there was a slightly significant decrease in taste acceptability when MOLP was increased from 5% to 10% in brown bread. In terms of taste acceptability, brown bread containing 5% MOLP has significantly higher taste acceptability in comparison to white bread containing 5% MOLP (*p* < 0.05). The colour acceptability significantly decreased when MOLP was added to white bread. There was no significant effect when 5% MOLP was added to brown bread (*p* > 0.05). However, there was a significant decrease in colour acceptability when 10% MOLP was added to brown bread. Brown bread containing 5% MOLP had a significantly higher colour acceptability compared to white bread containing either 5% or 10% MOLP and brown bread containing 10% MOLP.

As MOLP was added to both white and brown breads, respectively, there was a significant decrease in aroma acceptability (*p* < 0.05) and there was a slightly significant decrease when MOLP was increased from 5% to 10% in both white and brown breads. The texture acceptability significantly decreased when MOLP was added to white bread; however, there was no significant difference between white bread containing 5% and 10% MOLPs. The appearance acceptability decreased when MOLP was added to white and brown breads. Additionally, when MOLP was increased to 10%, there was a further significant decrease in appearance acceptability seen for white bread. The overall acceptability significantly decreased when MOLP was increased (*p* < 0.05)—this was especially seen for brown bread. With this being said, brown bread containing 5% MOLP had a significantly higher overall acceptability in comparison to the other MOLP-containing bread.

## 4. Discussion

The bread samples became a darker colour when MOLP was included in the dough; this was particularly prominent in white bread samples (Table 2, Figure 2). This was an expected result as *Moringa oleifera* leaves are naturally a dark green colour due to the high chlorophyll content [22] and are thus responsible for the undesirable colour change. The brown bread prepared in this study became a darker colour but was not as noticeable as white bread, and this could be due to the fact that brown bread is a darker colour to start with due to the chocolate colour of the bran [28], masking the undesirable darker colour seen in white bread at the same concentration of MOLP. These results were consistent with a study conducted by Bourekoua et al. (2018), which found that, as the MOLP concentration increased, the lightness of bread crumb and crust decreased [25]. This dark colour may negatively affect consumer acceptability of bread fortified with MOLP as consumers are more accustomed to bread being a golden-brown colour. With this being said, more individuals are will to try a product that they are familiar with if it was beneficial to their health, thus bread containing MOLP may be acceptable despite being a darker colour. White bread is more commonly consumed than brown bread, therefore another solution to this could be to use a lightening agent to mask the dark colour [23], thus making the bread containing MOLP more acceptable. The use of lightening agents was not investigated in this study. Brown bread containing 5% MOLP had a similar colour to the control thus implies that fortifying with a concentration lower than 5% may result in lighter bread with all the nutritional benefits.

Although MOLP has an undesirable physical attribute, as mentioned earlier, it has many nutritional benefits. Table 3 indicates that the protein concentration increased when MOLP was added to white and brown breads. White bread containing 10% MOLP had the highest protein content (15.5 g/100 g). This was expected as Moringa leaves have a high protein content [34]. The study results were similar to another study which found unfortified bread had the lowest protein content (8.5%) and bread fortified with MOLP contained the highest protein content (13.5%) [24]. Similarly, other authors found that there was a gradual increase in protein content as MOLP was added at different concentration levels [27]. Furthermore, said study had a higher protein content than the current study when 5% MOLP was added to the bread (17.72%).

Table 4 shows results of assessing the potential contribution of MOLP-supplemented bread to the EAR for protein for vulnerable population groups if the amount equivalent to the usual portion size of standard was consumed. It was found that all bread samples containing MOLP would contribute to meeting more than 50% of the EAR for protein for each of the vulnerable groups. Moreover, white bread containing MOLP would contribute more to meeting the EAR for protein for all vulnerable population groups. To fully meet the EAR for protein, a 1–3-year-old would need to consume three slices of the MOLP-containing bread/day. A 4–5-year-old would need to consume 4.5 slices of white bread containing MOLP/day or five slices of brown bread containing MOLP/day. The 14–18 year female group would have to consume 9.5 slices of white bread containing 5% MOLP/day and nine slices of white bread containing 10% MOLP/day and ten slices of brown bread containing MOLP/day. Lastly, the 19–50 year female group would need to consume ten slices of bread containing 5% MOLP/daily and 9.5 slices of white bread containing 10% MOLP. In contrast, 10.5 slices of brown bread containing MOLP would need to be consumed daily. The fact that MOLP increases the protein content is encouraging as animal sources of protein are good but expensive and not affordable to many. The high protein content found in bread containing MOLP could assist in reducing PEM. However, in order not to promote a monotonous diet focused on MOLP bread, MOLP should be incorporated in other commonly consumed food items that are also deficient in protein.

The iron concentration increased in white bread when 10% MOLP was added, and when 5% MOLP was added to brown bread (Table 3). This was an expected result as MOLP generally contains a high iron content. The iron values obtained in the current study agree with the results obtained from previous studies, which showed an increase in iron content as MOLP was added [23,24,27].

Analysis of the percentage contribution of MOLP-containing bread samples to the EAR value for iron for each of the vulnerable population groups is presented in Table 5. It was found that consumption of MOLP-containing bread, up to an amount equivalent of the usual portion size of standard bread, would result in the achievement of more than 100% of the EAR value for iron for each of the vulnerable groups. The iron intake, of each age group, that would result from consumption of MOLP-containing bread, up to an amount equivalent to the usual portion size of standard bread, was also compared with the Tolerable Upper Intake Level (UL). UL refers to the highest amount of a nutrient that should be consumed from food without any adverse effects [33]. The results show that the iron intake of 1–5-year-old children would be way below the UL (40 mg/d) if they consumed MOLP-containing bread up to an amount equivalent to the usual portion size of standard bread. For the 14–18 year and 19–50 year female groups, consumption of all MOLP-containing bread types (except for the brown bread containing 10% MOLP), up to an amount equivalent to the usual portion size of standard bread, would result in an iron intake below the UL value. It is noted that, even for the MOLP-containing bread types that would result in an iron intake above the UL value, it is unlikely to be of health concern because of the limited bioavailability of divalent metal minerals such as iron in plant-based foods. Overall, the results suggest that bread containing MOLP could be a cheaper alternative to improve the iron intake in vulnerable population groups.

Overall, consumer acceptability was higher in the control white and brown breads (0%) compared with their respective bread samples containing MOLP (Table 6 and Table 7). This could be lower due to the bitter taste [7] and the fact that it causes the colour of the product to become darker. These results concurred with other studies which found a decrease in overall acceptability with an increase in MOLP [25,27]. There was no significant difference in the instrument texture values of the bread samples. However, the texture acceptability of the bread samples decreased significantly in white bread with the addition of MOLP (*p* < 0.05), whilst there was a marginal difference in the texture acceptability of the control brown bread and bread samples containing MOLP. Thus, it appears that MOLP had a negative effect on the texture of white bread, as perceived by the consumers. MOLP increases the hardness of bread, which is likely due to its high fibre content, but the increase in hardness was perceived in white bread and not in brown bread, probably due to the fact that brown bread already has a high concentration of fibre compared to white bread.

The overall acceptability of brown bread containing 5% MOLP was higher than that of the white bread containing 5% MOLP, indicating that brown bread would be more suitable for fortification using MOLP compared to white bread. For consumers who prefer white bread to brown bread, the darkness imparted to the white bread by MOLP, which was disliked by the consumers, could be resolved by adding a lightening agent together with the MOLP to the white bread dough.

## 5. Conclusions

Protein-energy malnutrition and iron deficiency anaemia are public health concerns. A food-based intervention such as fortification of bread with MOLP could improve the protein and iron contents of bread. The results of the current study indicate that bread supplemented with about 5% MOLP could be used to complement existing strategies for addressing malnutrition, especially PEM. However, further research needs to be conducted in order to improve the physical attributes of bread containing MOLP. Further research involving incorporating MOLP in other popular, but nutrient-deficient foods, needs to be conducted to determine the most suitable foods for fortifying with MOLP.

## Figures and Tables

**Table 1:** Studies conducted on the addition of *Moringa* to bread.

Authors	Study Methods	Location	Participants	Findings
Bolarinwa, Aruna and Raji (2019) [24]	Bread was fortified with 0%, 5%, 10%, 15% and 20% Moringa seed powders. The proximate, mineral and vitamin A contents were determined. A sensory evaluation was conducted using a seven-point hedonic scale to assess consumer acceptability for the sensory attributes—colour, shape, texture, sweetness, flavour, mouthfeel and overall acceptability.	Nigeria	Twenty randomly selected judges.	Study results indicated the as Moringa seed powder was added to bread, there was a significant increase in protein, ash, fat, fibre, phosphorus, potassium, calcium, iron and vitamin A contents. However, there was a decrease in moisture and carbohydrates. The sensory evaluation results indicated that there was no difference between the control bread and the 5% Moringa fortified bread. Further, the bread containing 5% Moringa seed powder was rated the best for the sensory attributes investigated.
Obichili and Ifediba (2019) [28]	Experimental research study. Two samples were prepared. The authors prepared a whole wheat bread (control) and a whole wheat bread fortified with Moringa leaf powder (MLP) using the ratio 1:4 (*v*/*v*). A nine-point hedonic scale was used to assess the sensory attributes—colour, taste, flavour, texture and general acceptability.	Nigeria	Thirty-seven evaluators categorised into two groups of seven lecturers and 30 registered postgraduate students in the Department of Home Economics, University of Nigeria Nsukka.	The whole wheat bread containing MLP was rated better for the sensory attributes flavour, taste and general acceptability. In contrast, the whole wheat bread (control) was rated better for colour and texture.
Bourekoua, Różyło, Gawlik-Dziki et al. (2018) [25]	Gluten-free bread (made with rice semolina) was fortified with 2.5%, 5%, 7.5% and 10% MLP. Antioxidant activity was determined. The sensory acceptability was determined using a nine-point hedonic scale. The following attributes were assessed—taste, aroma, texture, appearance and overall acceptability.	Poland	Fifty-two untrained consumers (23–48 years, 28 females and 24 males)	There was a significant decrease in the volume of the bread samples as MLP was added with the exception of 2.5% MLP. With the addition of 2.5% and 10% MLPs, there was a slight decrease in hardiness and chewiness. The lightness of the bread decreased when MLP was added. As MOLP was increased from 0% to 10%, so too was the antioxidant activity. In comparison to the control, the most acceptable MLP-containing bread was with the addition of 2.5% MLP.
El-Gammal, Ghoneim and ElShehawy (2016) [27]	Pan bread was fortified with 5%, 10%, 15% and 20% MLPs. The moisture, fat, ash, crude fibre, magnesium, calcium, copper, zinc and iron contents were determined using standard methods. Sensory evaluation was conducted using the AACC method to access the smoothness, crust colour, crumb colour, taste and overall acceptability. Texture and stalling of the bread were also determined.	Egypt	Fifteen staff members from the Food Industries Department, Faculty of Agriculture, Mansoura University	The Moringa leaf powder contained a high protein, crude fibre, calcium, magnesium, phosphorous and iron content. The pan bread containing MLP had a high protein content but lower carbohydrate content in comparison to the control. Further, the 10% MLP pan bread had higher calcium, magnesium and iron contents compared to the control pan bread. The sensory acceptability decreased as the MLP concentration increased, especially with the 15% and 20% MLPs pan bread. When MLP was increased, there was a decrease in gumminess, chewiness and springiness and a gradual increase in freshness.
Sengev, Abu and Gernah (2013) [23]	Bread was prepared using 0%, 1%, 2%, 3%, 4% and 5% MLP. Moisture, crude protein, crude fat, crude fibre and ash content were determined according to standardised methods. The volume and weight of the baked loaves were determined. Meilgaard’s procedure was used to determine the sensory evaluation after 24 h. The sensory attributes of the bread that were evaluated were crust colour, crumb colour, crumb texture, flavour and overall acceptability.	Nigeria	Was not provided	There was a significant increase in the protein, fibre, ash, magnesium, calcium and beta-carotene content and a decrease in iron and copper content as the MLP concentration increased in the bread samples. The volume and loaf height decreased while the weight of the loaf containing MLP increased. There was a negative effect on the sensory evaluation as the MLP concentration increased.
Ogunsina, Radha and Indrani (2011) [26]	The bread samples were prepared using 0%, 5%, 10% and 15% dry Moringa oliefera seed flours. The nutritional contents (moisture, crude fat, as, crude protein, crude fibre, iron and calcium) were determined. A quantitative descriptive analysis was used. The sensory evaluation was conducted using a quantified structure scale (crust colour (10); shape (15) and symmetry (15); crumb colour (10); grain (20); mouthfeel (20) and taste (10)).	India	Suitably trained panellists. The total number of panellist was not provided.	This study found that bread fortified with 10% Moringa was acceptable. Additionally, the 10% Moringa fortified bread had higher protein, iron and calcium contents compared to the other samples.

**Table 2:** The effect of *Moringa oleifera* leaf powder fortification on colour and texture of white and brown breads.

Bread Samples (% *w*/*w* MOLP Added)	Colour and Texture (Mean ± SD)			
	L (Lightness)	a (Redness)	b (Yellowness)	Texture (g)
**White bread**				
0% (control)	67.14 ± 0.77 ^e^	1.56 ± 0.05 ^a^	24.1 ± 0.1 ^b^	0.09 ± 0.02 ^a^
5%	54.02 ± 0.14 ^b,c^	0.86 ± 0.02 ^a^	27.4 ± 0.1 ^d^	0.08 ± 0.01 ^a^
10%	45.97 ± 0.29 ^a^	1.31 ± 0.05 ^a^	25.4 ± 0.2 ^c^	0.13 ± 0.07 ^a^
**Brown bread**				
0% (control)	63.1 ± 0.6 ^d^	3.02 ± 0.07 ^b^	21.5 ± 0.3 ^a^	0.09 ± 0.02 ^a^
5%	55.8 ± 0.4 ^c^	2.67 ± 0.19 ^b^	24.9 ± 0.2 ^c^	0.08 ± 0.02 ^a^
10%	51.7 ± 2.0 ^b^	2.79 ± 0.87 ^b^	27.5 ± 0.2 ^d^	0.08 ± 0.03 ^a^

Different letters in columns show significant difference according to the Bonferroni test (*p* < 0.05).

**Table 3:** Nutritional composition of white and brown breads containing different concentrations of *Moringa oleifera* leaf powder (MOLP) on a dry weight basis (DW)].

Bread Samples (% MOLP Added)								
	Moisture g/100 g	Ash g/100 g	Fat g/100 g	NDF g/100 g	Protein g/100 g	Calcium mg/kg	Zinc mg/kg	Iron mg/kg
**White bread**								
0% (control)	35.18 ± 1.32 ^a^	1.97 ± 0.06 ^a^	3.12 ± 0.11 ^a^	13.75 ± 0.10 ^a^	13.68 ± 0.01 ^c^	0.04 ± 0.00	3.00 ± 0.00 ^a^	6.50 ± 2.83 ^a^
5%	31.84 ± 0.25 ^a^	2.40 ± 0.00 ^b,c^	3.58 ± 0.06 ^a^	19.02 ± 2.73 ^a^	13.96 ± 0.00 ^d^	0.10 ± 0.00	3.50 ± 9.90 ^a^	7.60 ± 2.83 ^a^
10%	32.39 ± 0.40 ^a^	2.54 ± 0.15 ^b,c^	3.33 ± 0.32 ^a^	14.77 ± 2.94 ^a^	14.59 ± 0.01 ^e^	0.15 ± 0.00	2.95 ± 0.71 ^a^	16.55 ± 2.12 ^b^
**Brown bread**								
0% (control)	33.56 ± 0.40 ^a^	2.28 ± 0.01 ^a,b^	2.98 ± 0.42 ^a^	23.87 ± 4.86 ^a^	13.07 ± 0.06 ^a^	0.03 ± 0.00	3.20 ± 0.00 ^a^	16.50 ± 2.83 ^b^
5%	38.93 ± 6.93 ^a^	2.46 ± 0.06 ^b,c^	2.78 ± 0.02 ^a^	20.60 ± 1.20 ^a^	13.16 ± 0.01 ^ab^	0.05 ± 0.00	3.05 ± 0.71 ^a^	23.20 ± 2.83 ^c^
10%	33.60 ± 0.10 ^a^	2.62 ± 0.04 ^c^	3.36 ± 0.13 ^a^	17.75 ± 1.14 ^a^	13.34 ± 0.10 ^b^	0.09 ± 0.00	3.10 ± 0.00 ^a^	30.95 ± 9.19 ^d^

Data reported as Mean ± SD of at least two replicates. NDF: Neutral detergent fibre; Different letters in columns show significant difference according to the Tukey test (*p* < 0.05).

**Table 4:** Percentage of the estimated average requirement met for protein for vulnerable population groups using the estimated portions of bread.

Bread Samples (% MOLP ^a^ Added)	1–3 Years			4–5 Years			14–18 Years (Female)			19–50 Years (Female)		
	Protein (g)	EAR ^b^ g/day	% EAR Met	Protein (g)	EAR g/day	% EAR Met	Protein (g)	EAR g/day	% EAR Met	Protein (g)	EAR g/day	% EAR Met
**White bread**												
0% (control)	6.15	11.31	54.4	12.30	18.24	67.4	24.60	39.05	63.0	36.90	40.92	90.2
5%	6.29	11.31	55.6	12.57	18.24	68.9	25.14	39.05	64.4	37.71	40.92	92.2
10%	6.57	11.31	58.1	13.14	18.24	72.0	26.28	39.05	67.3	39.42	40.92	96.3
**Brown bread**												
0%	5.88	11.31	52.0	11.76	18.24	64.8	23.52	39.05	60.2	35.28	40.92	86.2
5%	5.93	11.31	52.4	11.85	18.24	65.0	23.70	39.05	60.7	35.55	40.92	86.9
10%	6.00	11.31	53.1	12.00	18.24	65.8	24.00	39.05	61.5	36.00	40.92	88.0

^a^*Moringa oleifera* leaf powder; ^b^ Estimated average requirement (EAR) [33].

**Table 5:** Percentage of the estimated average requirement met for iron for vulnerable population groups using the estimated portions of bread.

Bread Samples (% MOLP ^a^ Added)	1–3 Years			4–5 Years			14–18 Years (Female)			19–50 Years (Female)		
	Iron (mg)	EAR ^b^ mg/day	% EAR Met	Iron (mg)	EAR mg/day	% EAR met	Iron (mg)	EAR mg/day	% EAR met	Iron (mg)	EAR mg/day	% EAR Met
**White bread**												
0% (control)	2.93	3.00	98.3	5.85	4.10	142.7	11.7	7.90	148.1	17.6	8.10	217.3
5%	3.42	3.00	114.0	6.84	4.10	166.8	13.7	7.90	173.4	20.5	8.10	253.1
10%	7.46	3.00	248.7	14.91	4.10	363.7	29.8	7.90	235.4	44.7	8.10	551.9
**Brown bread**												
0%	7.43	3.00	247.7	14.85	4.10	362.2	29.7	7.90	375.9	44.6	8.10	550.6
5%	10.44	3.00	348.0	20.88	4.10	509.3	41.8	7.90	529.1	62.6	8.10	772.8
10%	13.91	3.00	463.7	27.81	4.10	678.3	55.6	7.90	703.8	83.4	8.10	1029.6

^a^*Moringa oleifera* leaf powder; ^b^ EAR (Estimated average requirement) [33].

**Table 6:** The effect of *Moringa oleifera* leaf powder (MOLP) on the overall acceptability of white and brown breads as shown by percentage distribution of sensory evaluation scores.

Bread Samples (% MOLP Added)	Overall Acceptability										
	Dislike Extremely (1)	Dislike Very Much (2)	Dislike Moderately (3)	Dislike Slightly (4)	Neither Like Nor Dislike (5)	Like Slightly (6)	Like Moderately (7)	Like Very Much (8)	Like Extremely (9)	Overall Like (6–9)	Overall Dislike (1–4)
**White bread**											
0 (Control)	1 ^a^ (1.9) ^b^	1 (1.9)	0 (0)	1 (1.9)	7 (13.0)	10 (18.5)	9 (16.7)	13 (24.1)	12 (22.2)	44 (81.5)	3 (5.6)
5	3 (5.6)	4 (7.4)	4 (7.4)	8 (14.8)	10 (18.5)	9 (16.7)	9 (16.7)	5 (9.3)	2 (3.7)	25 (46.3)	19 (35.2)
10	7 (13.0)	6 (11.1)	8 (14.8)	12 (22.2)	7 (13.0)	5 (9.3)	4 (7.4)	2 (3.7)	3 (5.6)	14 (25.9)	33 (61.1)
**Brown bread**											
0 (Control)	0 (0)	0 (0)	0 (0)	0 (0)	3 (5.6)	9 (16.7)	10 (18.5)	16 (29.6)	16 (29.6)	51 (94.4)	0 (0)
5	1 (1.9)	2 (3.7)	1 (1.9)	3 (5.6)	10 (18.5)	7 (13.0)	13 (24.1)	8 (14.8)	9 (16.7)	37 (68.5)	7 (13.0)
10	2 (3.7)	4 (7.4)	2 (3.7)	7 (13.0)	14 (25.9)	9 (16.7)	10 (18.5)	3 (5.6)	3 (5.6)	25 (46.3)	15 (27.8)

^a^ number of panellists who gave the score; ^b^ percentage of the consumer panel (N = 54) that gave the score.

**Table 7:** The effect of *Moringa oleifera* leaf powder (MOLP) on sensory acceptability of white and brown breads.

Bread Samples (% MOLP Added)	Sensory Acceptability (Mean ± SD)					
	Taste	Colour	Aroma	Texture	Appearance	Overall Acceptability
**White bread**						
0 (Control)	6.28 ± 2.16 ^c,d^	7.46 ±1.40 ^c^	7.07 ± 1.52 ^c^	6.32 ± 2.04 ^b^	7.2 ± 1.59 ^c^	6.98 ± 1.79 ^cd^
5%	4.57 ± 2.07 ^a^	5.06 ± 2.29 ^a,b^	4.70 ± 2.31 ^a,b^	5.11 ± 2.34 ^a^	5.4 ± 2.31 ^b^	5.19 ± 2.07 ^ab^
10%	3.83 ± 2.25 ^a^	4.09 ± 2.48 ^a^	4.07 ± 2.26 ^a^	5.11 ± 2.12 ^a^	4.1 ± 2.70 ^a^	4.20 ± 2.23 ^a^
**Brown bread**						
0 (Control)	7.07 ± 1.46 ^d^	7.57 ± 1.11 ^c^	7.24 ± 1.36 ^c^	6.85 ± 1.89 ^b^	7.5 ± 1.33 ^c^	7.61 ± 1.23 ^d^
5%	5.91 ± 2.10 ^b,c^	6.56 ± 1.86 ^c^	5.74 ± 1.99 ^b^	6.06 ± 2.01 ^ab^	6.4 ± 2.07 ^b,c^	6.44 ± 1.96 ^c^
10%	4.78 ± 2.06 ^a,b^	5.44 ± 2.07 ^b^	4.85 ± 1.99 ^a,b^	5.87 ± 1.82 ^a,b^	5.7 ± 2.06 ^b^	5.35 ± 1.94 ^b^

Means marked with different letters in the same column are significantly different at *p* < 0.05 according to the Tukey test.
